# Human Cytomegalovirus Induces TLR4 Signaling Components in Monocytes Altering TIRAP, TRAM and Downstream Interferon-Beta and TNF-Alpha Expression

**DOI:** 10.1371/journal.pone.0044500

**Published:** 2012-09-07

**Authors:** Kok-Hooi Yew, Cory Carpenter, R. Scott Duncan, Christopher J. Harrison

**Affiliations:** 1 Pediatric Infectious Disease, Children’s Mercy Hospitals, Kansas City, Missouri, United States of America; 2 Department of Pediatrics, University of Missouri School of Medicine, Kansas City, Missouri, United States of America; 3 Kansas City University of Medicine and Biosciences, Kansas City, Missouri, United States of America; University of Leuven, Rega Institute, Belgium

## Abstract

Using TLR pathways, primary human cytomegalovirus (HCMV) induces innate responses including the production of inflammatory cytokines. Mounting evidence suggests that LPS recognition by TLR4/MD2/CD14 results in differential utilization of TIRAP-TRAF6 and TRAM-TRIF signaling, thereby leading to transcriptional activation of various cytokine genes. However, relative roles of the TLR4/MD2/CD14 complex and its adaptor proteins TIRAP and TRAM involved in regulating monocyte responses to HCMV are incomplete. Here, we provided evidence supporting the notion that the TLR4/MD2/CD14 complex contributes notably to HCMV-induced signaling and subsequent cytokine production in monocytes. In particular, induction of both IL-6 and IL-8 is associated with elevated TIRAP and reduced TRAM mRNA expression. The latter may serve in a compensatory pathway that yields a robust IFN response when TIRAP signaling is blocked in monocytes incubated with Toledo strain HCMV. Inhibitory studies using antisense oligonucleotides or neutralizing antibodies indicate that IL-6 induction by TLR4/MD2 complex is important for the activation of endogenous CD14 which later acts in concert or synergy with TLR4/MD2 as a factor resulting in IL-8 gene expression. We further show that exogenous recombinant CD14 can potentiate innate immune response via TLR4-dependent and possibly via TLR9-dependent pathways to promote enhanced expression/production of IL-8 and IFN-β, respectively.

## Introduction

Toll like receptors (TLRs) are an evolutionary conserved family of type 1 membrane receptors that are required for sensing the presence of microorganisms and trigger inflammatory responses, such as cytokine release [1]. To date, ten human TLRs (TLR1 to TLR10) have been identified and characterized. This repertoire of TLRs mediates recognition and inflammatory responses to a broad spectrum of microbial and viral products [2] and is crucial for effective host defense aimed at control of the invading pathogens. Of these, TLR4 has widely been shown to be the signal-transducing receptor activated by bacterial lipopolysaccharide (LPS). This finding led to the moniker for TLR4 as ‘the LPS receptor’ [2], [3]. In addition to TLR4, two accessory molecules, MD2 and CD14, are also essential for LPS-induced TLR4 response [3], [4].

Downstream signaling via TLR4 originates from its conserved cytoplasmic domain, the TIR domain. It is peculiar among other TLRs in its ability to facilitate the engagement of two distinct TIR domain-containing adaptor proteins: TIRAP (also known as Mal), which recruits MyD88 and TRAM (also called TICAM2 or TIRP), which recruits TRIF [5]. The MyD88-TIRAP (MyD88 dependent) complex activates TRAF6 via IRAK kinases, whereas the TRAM-TRIF (MyD88 independent) module recruits RIP1 or TRAF6 [6], leading to the induction of IL-6 and IL-8 [7] as well as IFN-β [6], respectively. These cytokines themselves appear to play an important role in the pathogenesis of HCMV after bone marrow transplantation and can be useful predictors for HCMV infection and disease [8].

CD14 is a 55-kDa glycoprotein found in nature either in a membrane-bound form or a soluble form. Membrane-bound CD14 (mCD14) is attached to the membrane by a glycosylphosphatidylinositol (GPI) anchor which excludes direct signal transduction without the involvement of other membrane constituents [9]–[11]. Soluble CD14 (sCD14) lacks the GPI anchor but has the same amino acid sequence as mCD14. Both forms enhance cell responsiveness to various bacterial/viral products [3], [4], [10].

Another protein that has also been shown to be critical for the TLR4/CD14 interaction is the extracellular adaptor protein MD2. It is a small cysteine-rich glycoprotein that binds to the ectodomain of TLR4 in the endoplasmic reticulum and then transits to the cell surface in an active TLR4/MD2 complex [4], [9], [12]. Both co-receptors are equally important to stabilize TLR4 expression on the cell surface following engagement of TLR4 with its prototype ligand, LPS [1]–[4], [9].

Due to the conserved nature of the TLR4/MD2/CD14 complex, a growing number of reports suggest that the complex is biologically relevant and responsive to viral proteins, including those of Ebola virus [13] in monocytes, hepatitis C virus [14] in bone marrow cells and respiratory syncytial virus [15] in lung cells, thereby leading to induction of proinflammatory cytokines. Nevertheless, little is known about the mechanism by which the components of the TLR4/MD2/CD14 complex mediate such effects in monocytes when TLR4 signaling is induced by HCMV.

**Figure 1:**
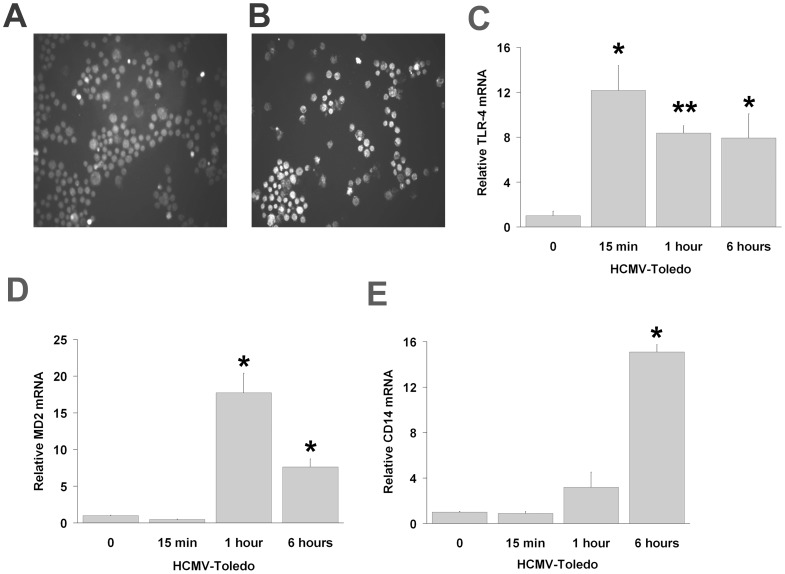
HCMV activates THP-1 cells via the TLR4/MD2/CD14 receptor complex. A and B: Immunostaining of THP-1 cells with and without HCMV stimulation for TLR4 showed marked upregulation of TLR4-positive staining. C–E: Quantitative gene expression analysis using real-time qPCR in TLR4, MD2, and CD14 reflected similar upregulation seen in A. All mRNAs were analyzed from the same preparation. All data are means±S.D. of experiments performed in triplicate. Reaction mixtures without reverse transcriptase served as controls for genomic DNA contamination in all cases (Data not shown).

Previous studies from other investigators have clearly determined the involvement and importance of TLR2 and CD14 in HCMV attachment, uptake and subsequent signaling leading to expression of pro-inflammatory cytokine genes [16], [17]. Likewise, TLR4 and endosomal TLRs, such as TRL3 and TLR 9, are also involved in HCMV-elicited signaling leading to a cellular pro-inflammatory and antiviral response [16], [17]. Thus HCMV appears to induce signaling via multiple TLRs, with characteristics that differ by being MyD88 dependent or independent as well as using TLRs that predominantly reside on cell surfaces or in endosomes. These data prompted us to further investigate the role of transduced molecules and modulatory factors within the signaling pathways of the TLR4/MD2/CD14 complex as an additional important signaling pathway during the immune response to HCMV.

The purpose of the current investigation is to identify aspects of signaling through TLR4 together with TLR4 accessory receptors/factors with which they interact, and to determine how HCMV may induce cytokine production from human monocytes. In testing our hypotheses, we utilized our well-established THP-1 monocytic cell induction model and employed a variety of molecular and cellular approaches to determine whether HCMV induces TLR4/MD2/CD14 complex signaling through the MyD88-dependent or the MyD88-independent pathway by assaying gene expression of selected but representative downstream signaling molecules and cytokines.

We first noted that HCMV induces THP-1 cells signaling via the TLR4/MD2/CD14 complex which initiates and regulates additional downstream signaling via TRAF6 in a TIRAP-dependent pathway, which in turn induces IL-6 and IL-8. We also showed that TRAM signaling was notable only with impaired TIRAP activity, thus possibly representing an “alternative” signaling route. This alternative pathway signaling also appears to increase activation of IFN-inducible genes via the TRAM-TRIF pathway, shifting from the canonical IL-6/IL-8 response to an IFN-β dominant response. We further observed that IL-6 induction by TLR4/MD2 is required for CD14 activation. This CD14 activation then acts in concert with TLR4/MD2 to enhance IL-8 mRNA transcription via a TIRAP-TRAF6-dependent pathway. Furthermore, exogenous recombinant CD14 restores the ability of IL-6-deficient THP-1 cells to augment IL-8 response through a TLR4-dependent pathway as well as enhancing IFN-β induction possibly via a TLR9-dependent mechanism.

**Figure 2:**
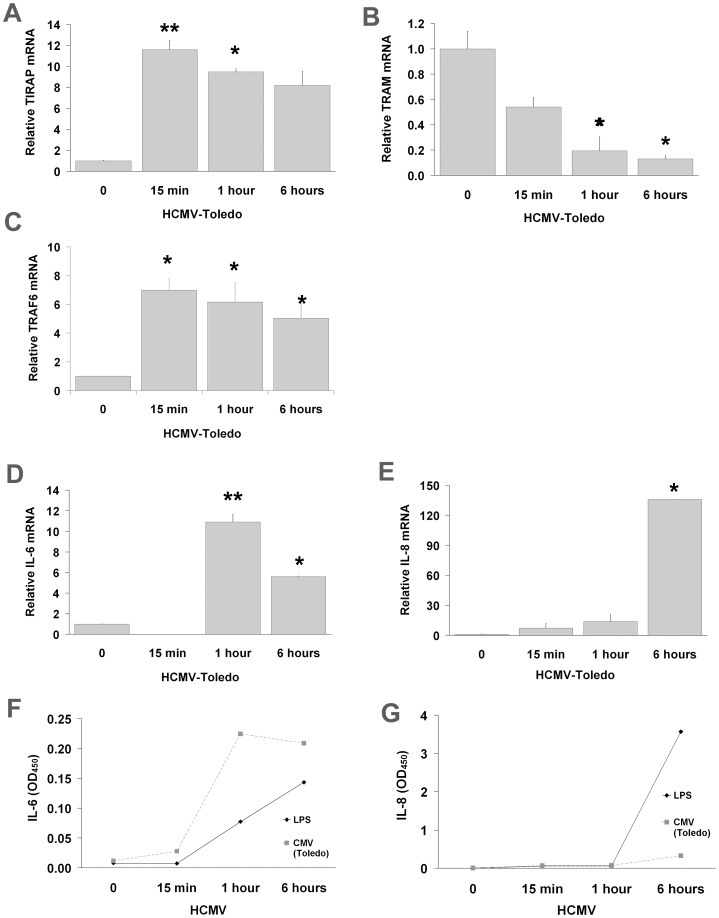
Quantitative analysis of TLR4-mediated signaling molecules and cytokine transcriptions in THP-1 cells induced by HCMV. All data are means±S.D. of experiments performed in triplicate. A: TIRAP levels were seen to increase even at the earliest times after incubation with HCMV. B: TRAM conversely showed a marked reduction of mRNA levels in response to HCMV, particularly at 6 hours. C: TRAF6, which potentially serves as a rate-limiting factor, was not found to be significantly altered at any time point. D and E: TLR4 regulating cytokine IL-6 and IL-8 expressions were elevated at 1 hour and 6 hours, respectively. F and G: The production of IL-6 and IL-8 measured by ELISA correlated well with the results of D and E. *p<0.05; ** p<0.01 by student T-test.

## Results

### Toledo Strain HCMV Added to THP-1 Monocytic Cells Activates the TLR4/MD2/CD14 Complex

TLR4 engagement of diverse ligands (bacterial LPS and components of many viruses) in multiple cell types initiates formation of a TLR4/MD2/CD14-complex as an essential step in signaling; this induces production of inflammatory cytokines [1]–[4], [13]–[15] via the MyD88-dependent pathway or the MyD88-independent pathway [5], [6]. Unlike TLR2, there are surprisingly sparse data on HCMV-induced TLR4 signaling in monocytes. To examine the role of TLR4 receptor complex in THP-1 cells incubated with laboratory strain of HCMV that retains more wild-type genes than AD169 strain, we first used RT-PCR to evaluate a broad time-response of THP-1 cells cultured in the presence or absence of Toledo HCMV.

**Figure 3:**
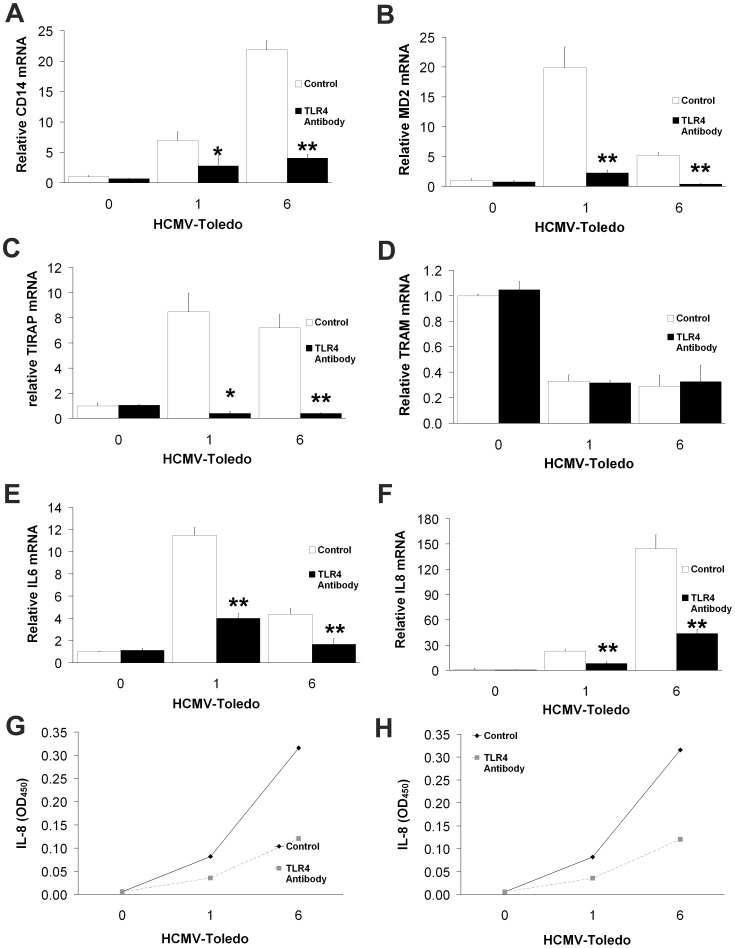
Effects of neutralizing TLR4 antibody compared to goat IgG control antibody on TLR4-induced gene transcription and cytokine activation. A: The increase in CD14 mRNA levels upon incubation with HCMV for 6 hours was significantly reduced by the TLR4 neutralizing antibody. B–F: Similar results were obtained for MD2 (B), TIRAP (C), IL-6 (E) and IL-8 (F). However, no effect on TRAM (D) was seen. (means±S.D. n = 4) (*p<0.05, **p<0.01 by students T test compared with control at 1 hour and 6 hours). G and H: ELISA analysis confirmed expected decreased levels of IL-6 (G) and IL-8 (H). All data are means of experiments performed in triplicate except where noted.

We determined that TLR4 upregulation via increased fluorescence intensity in the presence of HCMV (Fig. 1A & B) using immunocytochemical (ICC) staining. Using SYBR Green I real-time assays, we determined the precise time of upregulation for TLR4, CD14 and MD2 genes. We found that HCMV-induced levels of TLR4 mRNA in the THP-1 cells peaked early at 15 min and remained elevated throughout the incubation time (Fig. 1C). In contrast, MD2 and CD14 responses peaked later at 1 hour and 6 hours of incubation with HCMV, respectively (Fig. 1D and 1E).

In order to determine whether a brief incubation of THP-1 cells with HCMV results in actual infection, THP-1 cells were incubated with HCMV-Toledo for 1 and 6 hours followed by washing to clear residual HCMV in the media. Further incubation of these THP-1 cells for 3 days revealed that the initial brief incubations for leads to HCMV infection that can be detected by PCR (Fig. S1 A & B, respectively). In addition, we wanted to visualize HCMV immediate early antigen (IEA) and late antigen (LA) expression in infected THP-1 cells. This was achieved by exposing THP-1 cells to HCMV-Toledo for either 10 or 60 minutes followed by washing and incubation for 72 hours. After the 72 hour incubation period, cells were fixed and immunolabeled with antibodies to detect immediate early antigens (IEA) or late antigens (LA). Both exposure times resulted in positive immunoreactivity for IEA and LA (Fig. S1 C and D, respectively).

Other experiments in triplicate revealed 15–25% of THP-1 cells from these incubations are productively infected. Further, microscopic examination reveals a physical change in morphology from the non-adherent rounded form seen in unstimulated cells to an angular form that is plastic adherent in 65–85% of cells after 4 days of total incubation. These data suggest that among THP-1 cells incubated for short times (15 minutes, 1 and 6 hours) with a ratio of 10 virions per THP-1 cell, a small proportion are productively infected but a larger proportion are stimulated. It is likely therefore, that these brief incubations with HCMV resulted in HCMV contact with most if not all the THP-1 cells, but uptake of virions or viral components was likely by only a proportion of the co-incubated THP1 cells. It is the latter uptake of virions or viral components that may lead to ‘detection’ by intracellular/endosomal TLRs [16], [17], [18].

**Figure 4:**
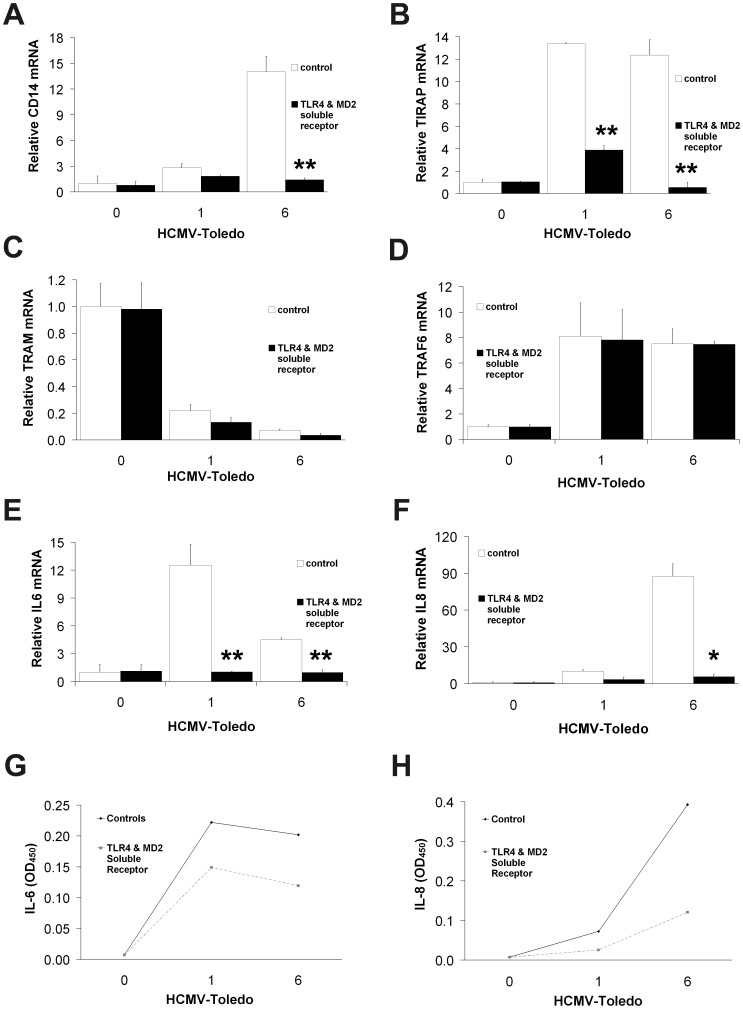
HCMV induced effects of recombinant TLR4/MD2 soluble receptors on THP-1 cells. CD14 mRNA (A) increases at 6 hours were blunted in the presence of TLR4/MD2 soluble receptors but not in media absent the exogenous soluble receptors, consistent with the results seen for neutralizing TLR4 antibody in Figure 3. Similar effects were seen on TIRAP (B), IL-6 (E) and IL-8 (F), suggesting that TLR4/MD2 complex signaling was essential for IL-6 and IL-8 induction. However, the soluble receptors had a minimal effect on TRAM (C) and TRAF6 (D). (means±S.D. n = 4). IL-6 (G) and IL-8 (H) secretion measured by ELISA showed similar findings. All data are means+S.D. of experiments performed in triplicate unless otherwise noted. *p<0.05; ** p<0.01 by student T-test.

### HCMV Up-regulates TIRAP/Mal Expression in THP-1 Monocytes

In light of the role of HCMV in inducing TLR4 receptor complex activation in our model (Fig. 1) and prior evidence of enhanced IL-6 and IL-8 cytokine responses in HCMV-IE transfected or HCMV-infected monocytes [19], [20], we next determined whether the TLR4-mediated activation of downstream adaptor molecules (TIRAP and TRAM) plays a regulatory role in the inflammatory cytokine response to HCMV in THP-1 cells. TIRAP is strongly upregulated as early as 15 minutes post THP-1 incubation with HCMV (Fig. 2A). Conversely, TRAM mRNA (Fig. 2B) was significantly decreased in THP-1 cells incubated with HCMV but not in monocytes incubated with virus free supernatants of human foreskin fibroblasts (HFF) cells. To determine whether either of these two adaptor molecules operates in parallel to TRAF6, (potentially acting as a rate-limiting factor), we assayed RNA levels of TRAF6, and found them more similar to TIRAP than to TRAM (Fig. 2C) at each assay point post incubation with HCMV. IL-6 expression in response to HCMV infection (Fig. 2D) was similar to that observed for MD2 expression (Fig. 1D), peaking at 1 hour post incubation with HCMV. In contrast, elevated IL-8 levels (Fig. 2E) were detected at six hours, similar to the pattern observed for CD14 (Fig. 1E). Levels of IL-6 (Fig. 2F) and IL-8 (Fig. 2G) proteins were found to correlate with the mRNA (Fig. 2D & 2E) as determined by ELISA.

**Figure 5:**
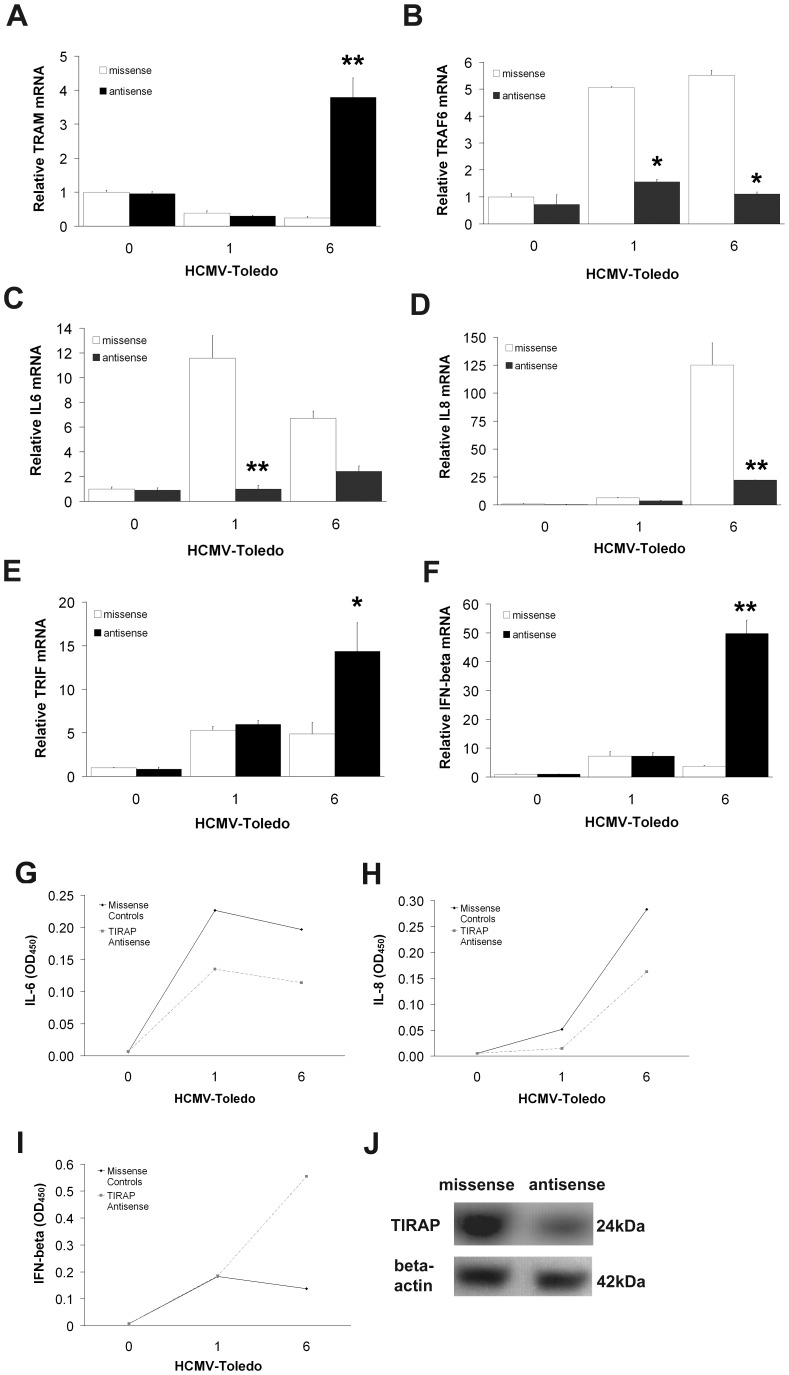
Role of TIRAP in TLR4-mediated regulation of cytokine gene expression in THP-1 cells induced by HCMV. A: TIRAP antisense treatment dramatically enhances levels of TRAM mRNA. B–D: Unlike previous elevations of TRAF6 mRNA (B), it was greatly inhibited by the TIRAP antisense. TIRAP-TRAF6-induced IL-6 (C) and IL-8 (D) mRNA elevation was also accompanied by a marked suppression. Interestingly, TRIF-induced (E) activation of IFN-beta (F) was markedly elevated by the TIRAP antisense, suggesting that a reversal exchange took place through the action of an alternative mechanism to activate IFN-inducible genes via the TRAM-TRIF pathway. (means±S.D. n = 4) (*p<0.05, **p<0.01 by student T-test compared with control at 1 hour and 6 hours) open squares = missense, closed squares = TIRAP antisense. The ELISA results showed an inhibition of both IL-6 (G) and IL-8 (H) releases whereas secretion of IFN-β was significantly enhanced. All data are means of experiments performed in triplicate except where noted. J: Western blotting confirms the inhibitory effects of the antisense on TIRAP levels; (n = 3). β-actin served as control for equal loading.

### Effects of TLR4 Blockade on HCMV-induced Cytokine Responses in THP-1 Cells

Prior data (Fig. 1) shows that incubation of THP-1 cells with HCMV activates TLR4 receptors at various times of incubation (15 min, 1 hour and 6 hours), together with upregulation of TLR4/MD2-mediated IL-6 and TLR4/CD14-mediated IL-8 transcription at 1 hour and 6 hours, respectively. This suggests that TLR4 may be a major mediator of HCMV-induced cytokine responses in THP-1 cells. To further verify the importance of TLR4 in HCMV-mediated cellular responses, we performed a similar set of experiments with a focus on 1 hour (IL-6) and 6 hours (IL-8) of THP-1 incubation with HCMV using an anti-human TLR4-neutralizing antibody as an antagonist. Initial experiments incubating TLR4 antibody with THP-1 cells showed no loss of viability after 4 days of incubation. Further, incubation of TLR4 antibody with THP-1 cells in the presence of poly I:C did not significantly impair interferon responses (data not shown).

**Figure 6:**
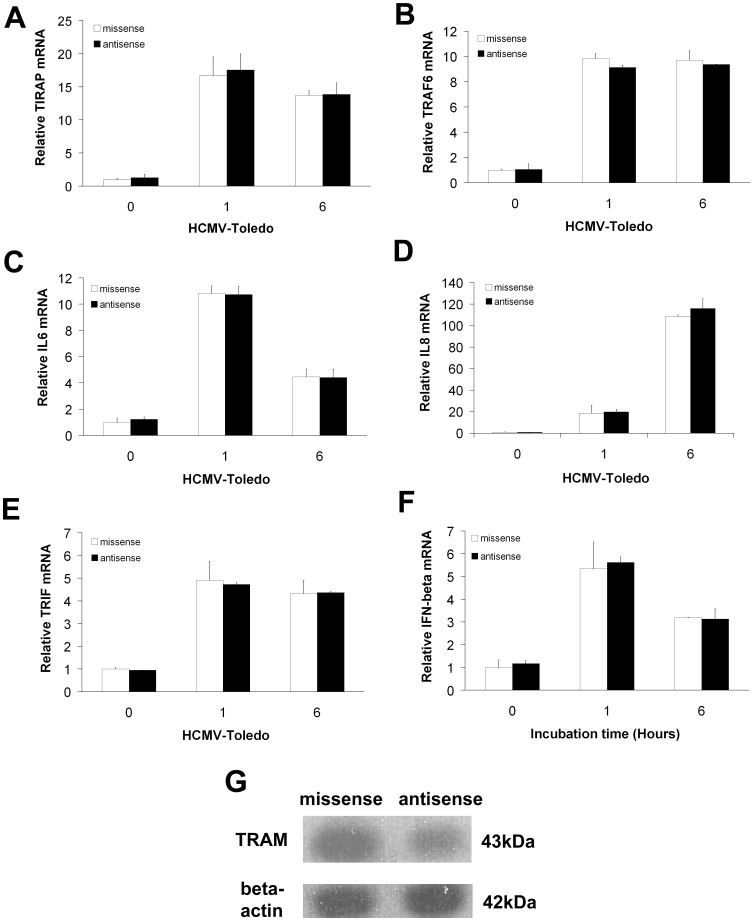
HCMV-induced effects of TRAM in TLR4-mediated regulation of cytokine gene expression in THP-1 cells. A–F: The mRNA levels of all of the key molecules remained stable in the presence of the TRAM antisense, which is the opposite result of what is seen with the TIRAP antisense, suggesting that TRAM may not be a critical regulator in signaling triggered by HCMV in THP-1 cells. Open squares = missense, closed squares = TRAM antisense. G: Western blot confirmed sequence-specific decreases in target TRAM by antisense. All data are means±S.D. of experiments performed in triplicate.

**Figure 7:**
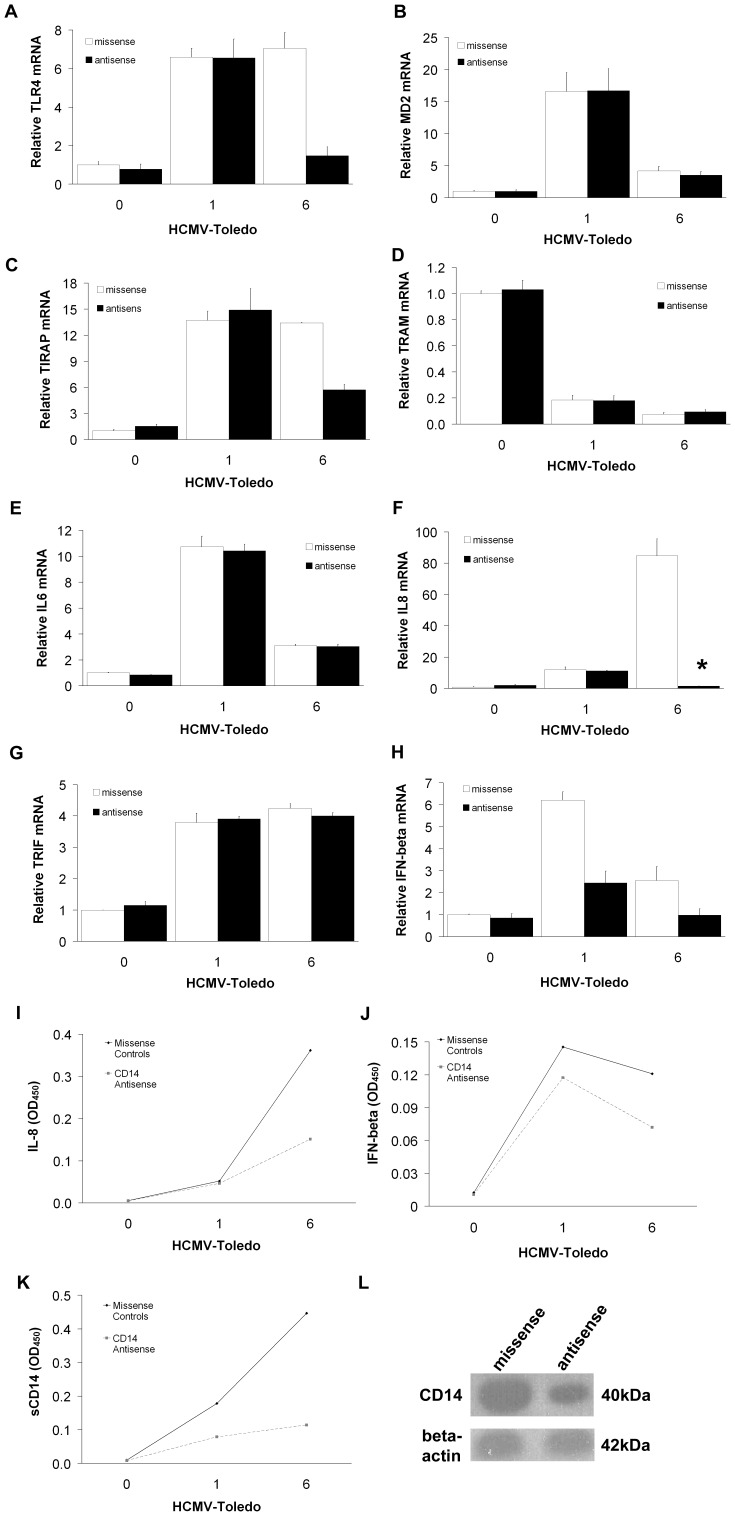
Role for CD14 in mediating CMV-induced cytokine expression. TLR4 (A) and TIRAP (C) mRNA elevations at 6 hours of HCMV co-incubation were suppressed in the CD14 antisense-treated THP-1 cells, but little effect was seen on TRAM (D) and MD2 (B). However, IL-8 (F) and IFN-β (H) were abolished by the CD14 antisense whereas IL-6 (E) and TRIF (G) were unaffected, suggesting that CD14 is the responsible receptor for inducing IL-8 and IFN-β transcription via the TLR4/TIRAP-dependent pathway and possibly the TLR9/MyD88-dependent pathway, respectively. (means±S.D. n = 4) (*p<0.05, **p<0.01 by Student T-test compared with control at 1 hour and 6 hours) open squares = missense, closed squares = CD14 antisense. ELISA analysis showed that the CD14 blockade reduced the in vitro production of IL-6 (I), IL-8 (J) and sCD14 (K). Western blot for CD14 confirmed sequence-specific effect of the antisense (L). (n = 3). All data are means of experiments performed in triplicate except where noted.

Anti-TLR4 treatment resulted in significant reduction of CD14 (Fig. 3A) and MD2 (Fig. 3B) mRNA expression in THP-1 cells incubated with HCMV. These results demonstrate that CD14 and MD2 are regulated by TLR4-dependent cell activation induced by HCMV. Furthermore, HCMV-induced increases in TIRAP mRNA (Fig. 3C) were dramatically reduced by TLR4 antibody. In contrast TLR4 blockade did not modify HCMV-induced decreases in TRAM (Fig. 3D) mRNA. This suggests that TLR4 is an important receptor inducing TIRAP transcription after HCMV-induced TLR4 activation. In contrast, TLR4 blockade strongly diminished IL-6 (Fig. 3E, G) and IL-8 (Fig. 3F, H) induction, suggesting that TLR4 is a contributor to HCMV’s role in stimulating production of these cytokines. These observations imply an important role for TLR4 in HCMV response via a TLR4-TIRAP-dependent signaling pathway.

**Figure 8:**
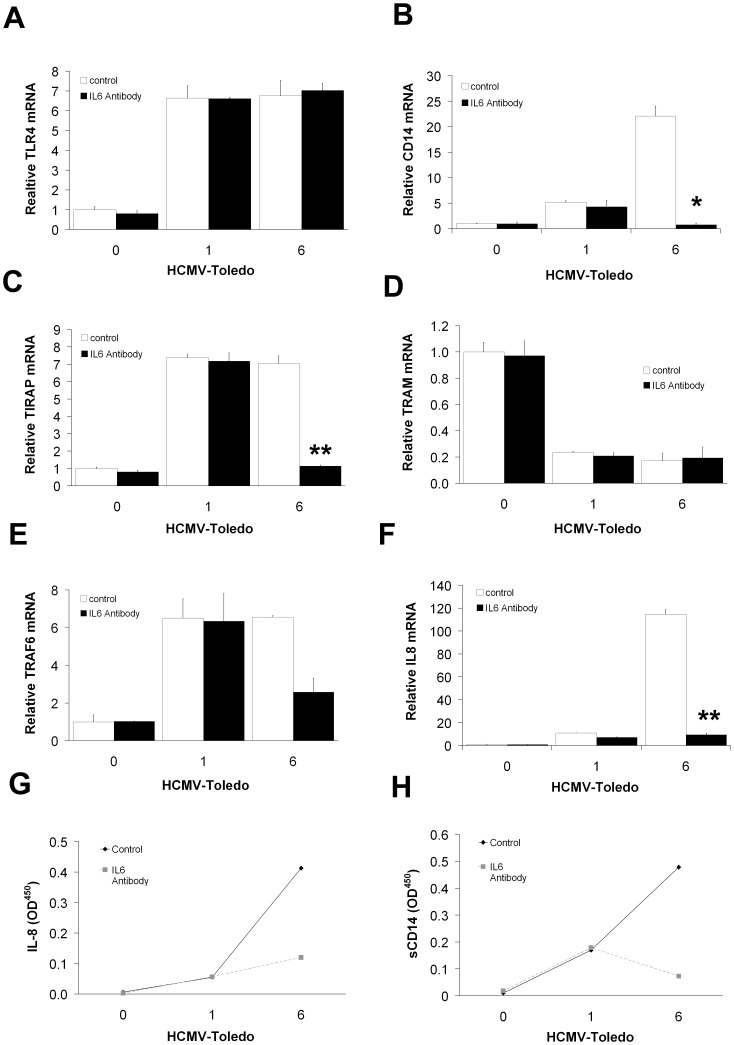
Role of IL-6 in THP-1 cells incubated with HCMV. No change was seen for TLR4 (A) and TRAM (D) in the presence of IL-6 antibody. However, the IL-6 antibody had an inhibitory effect on CD14 (B), TIRAP (C), TRAF6 (E) and IL-8 (F). (means±S.D. n = 4) (*p<0.05, **p<0.01 by student T-test compared with control at 6 hours). The secretion of cytokines IL-8 (G) and sCD14 (H) from IL-6-antibody-treated THP-1 cells was analyzed by ELISA and the results were similar to those observed by real-time PCR. Elisa data are means of experiments performed in triplicate.

### Soluble TLR4/MD-2 Receptors Competitively Inhibit HCMV-induced IL-6 and IL-8 in THP-1 Cells

Despite published evidence of LPS’ recognition by TLR4/MD2 [1]–[4], [9], little is known about HCMV interaction with TLR4/MD2. We sought to define the role of TLR4/MD2 complex in regulating HCMV-induced cytokine induction, particularly IL-6. We determined that IL-6 is differentially regulated by TLR4/MD2 (Fig. 2 and Fig. 3). First we tested whether TLR4/MD2 complex involvement in HCMV-mediated cytokine activation is similar to that previously shown for LPS-induced TLR4/MD2 signaling [5]–[7]. To evaluate induction of cytokine expression and production by HCMV in THP-1 cells, we used exogenous soluble recombinant TLR4/MD2 complex to compete with resident membrane-bound TLR4/MD2 complex. Soluble TLR4/MD2 complex suppressed HCMV-elicited CD14 mRNA expression (Fig. 4A), particularly at 6 hours. It also reduced TIRAP (Fig. 4B), but not TRAM (Fig. 4C) or TRAF6 (Fig. 4D) expression similar to our previous findings with TLR4 neutralizing antibodies (Fig. 3C). Exogenous recombinant soluble TLR4/MD2 inhibited both IL-6 (Fig. 4E and 4G) and IL-8 (Fig. 4F and 4H) expression and secretion.

**Figure 9:**
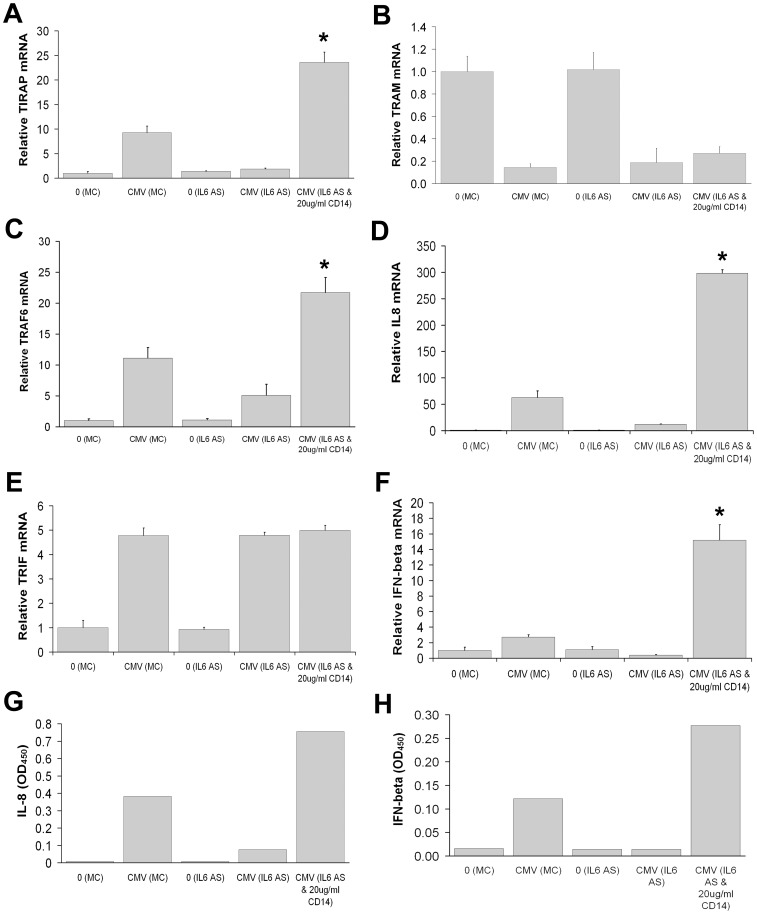
Rescue effects of exogenous recombinant CD14 in THP-1 cells incubated with HCMV and treated with IL-6 antisense. TIRAP (A), TRAF6 (C), and IL-8 (D) levels at 6 hours were up-regulated by the addition of 20 ug/ml CD14. Rescue of IFN-β (F) levels at 6 hours was also seen. However, rescued cells showed no change in TRAM (B) and TRIF (E) levels. (means±S.D. n = 4) (*p<0.05, **p<0.01 by student T-test compared with control at 6 hours). ELISA revealed a dramatic increase in IL-8 (G) and IFN-β (H) production. Elisa data are means of experiments performed in triplicate.

### TIRAP/Mal Inhibition Activates TRAM Transcriptional Regulation of TLR4-mediated Inflammatory Cytokine Induction

We determined that in our experiments noted above, TRAM expression is decreased in contrast to a large increase in TIRAP expression at both 1 and 6 hours after incubation with HCMV. In contrast, TIRAP antisense treatments of similarly HCMV-co-incubated THP-1 cells led to a large rise in TRAM expression at 6 hours (Fig. 5A), but had no effect on the expression of TLR4, CD14 and MD2 (data not shown). The previously noted up-regulation of TRAF6 mRNA levels at 1 and 6 hours (Fig. 5B) post incubation with HCMV was not observed after TIRAP antisense treatments. This suggests that TIRAP is critical for mediating TRAF6 transduction in THP-1 cells incubated with HCMV. Similar inhibiting effects were seen with TRAF6-induced expression and secretion of IL-6 (Fig. 5C and 5G) and IL-8 (Fig. 5D and 5H) at both 1 and 6 hours of HCMV co-incubation.

We next examined the effects of TIRAP-antisense treatments on TLR4-induced MyD88-independent TRIF and IFN-β expression in THP-1 cells incubated with HCMV. Interestingly, like TRAM, both TRIF (Fig. 5E) and IFN-β (Fig. 5F) levels were markedly elevated by TIRAP antisense treatments, particularly at 6 hours. Likewise, a robust IFN-β protein production (Fig. 5I) was detected at 6 hours by ELISA in TIRAP-depleted THP-1 cells co-incubated with HCMV. To confirm the inhibiting effects of the TIRAP antisense (Fig. 5J), Western blotting was performed in antisense-treated and sense-treated controls, confirming sequence-specific decreases in TIRAP by antisense.

**Table 1:** PCR Primer Sequences.

Genes	GenBank Accession #	Primer Sets	Product Size (bp)
B-actin	NM_001101	FP: AGAAAATCTGGCACCACACC	
		RP: GGGGTGTTGAAGGTCTCAAA	142
HCMV gB	X04606	FP: TGGTCTACAAGCGCAACATC	
		RP: GCCACGTATTCCGTATTGCT	130
TLR4	NM_138554	FP: CGGAGGCCATTATGCTATGT	
		RP: TCCCTTCCTCCTTTTCCCTA	141
CD14	NM_000591	FP: AGCCACAGGACTTGCACTTT	
		RP: TGGGCAATGCTCAGTACCTT	123
MD2	NM_15364	FP: CCGAGGATCTGATGACGATT	
		RP: TGGGCTCCCAGAAATAGCTT	142
TIRAP	AB446477	FP: TGGTGCAAGTACCAGATGCT	
		RP: GACTTGACGAAAGCCACCAT	162
TRAM	AY232653	FP: CTCTTGGGGTAAAAGGCACA	
		RP: TGTTGGCCCCTCTGTTGTAT	124
TRAF6	NM_145803	FP: TCGAACCCTTGAGGACAAAG	
		RP: CGGGTTTGCCAGTGTAGAAT	160
TRIF	AB093555	FP: ACGCCACTCCAACTTTCTGT	
		RP: TCAGGTGAGCTGAACAAGGA	136
IFN-β	M28622	FP: CATTACCTGAAGGCCAAGGA	
		RP: AGCAATTGTCCAGTCCCAGA	150
IL-6	NM_000600	FP: CTGCGCAGCTTTAAGGAGTT	
		RP: TAAGTTCTGTGCCCAGTGGA	135
IL-8	NM_000584	FP: AAGAAACCACCGGAAGGAAC	
		RP: ACTCCTTGGCAAAACTGCAC	123

(FP = Forward Primer; RP = Reverse Primer; bp = basepair).

### In Monocytes Co-incubated with HCMV, TRAM is not Involved in Canonical Signaling that Enhances IL-6/IL-8 or IFN-β Production

After noting that TIRAP antisense treatments increased TRAM expression and resultant IFN-β induction (Fig. 5), we sought to determine whether TRAM antisense treatment affected HCMV induction of TLR4-mediated MyD88-dependent signaling in THP-1 cells.

Neither TIRAP (Fig. 6A) nor TRAF6 (Fig. 6B) expression was modified by TRAM-antisense in THP-1 cells incubated with HCMV, suggesting that TRAM is not integrally involved in the regulation of the canonical HCMV-induced TLR4-mediated signaling pathway. We next used qPCR and ELISA to measure IL-6 and IL-8 expression (Fig. 6C and 6D) and secretion (data not shown) by TRAM-antisense or antisense-control treated THP-1 cells co-incubated with HCMV. Expression and secretion of both cytokines was unmodified by antisense treatment. Further, TRAM-antisense treatments did not modify activation of TRIF (Fig. 6E) or IFN-β (Fig. 6F) compared to the missense-control treatment. Together, these data indicate that neither the MyD88-dependent nor the MyD88-independent cytokine responses, when induced via the HCMV-induced canonical TLR4 signaling pathways, are directly affected by TRAM signaling. The effectiveness of the antisense in inhibiting TRAM protein is shown in the Western blot with suppression of TRAM in the presence of TRAM antisense (Fig. 6G).

### The CD14 Molecule is Important for IL-8 and IFN-b Gene Expression and Secretion Induced by HCMV in THP-1 Cells

Since the more pronounced IL-8 response of THP-1 cells to incubation with HCMV seemed to be associated with the higher expression of CD14 molecule at 6 hours, we examined the effect of CD14 antisense treatment on IL-8 expression and production. CD14 antisense reduced the expression of TLR4 (Fig. 7A) and TIRAP (Fig. 7C) at 6 hours of co-incubation with HCMV as well as the expression (Fig. 7F) and secretion (Fig. 7I) of IL-8. In contrast, addition of CD14 antisense had no substantial effect on the activation of MD2 (Fig. 7B), TRAM (Fig. 7D) or IL-6 (Fig. 7E). This suggests that CD14 and TLR4 are important in TRAF6-mediated TIRAP-induced IL-8 production. Moreover, increased TRIF mRNA (Fig. 7G) in THP-1 cells, pre-treated withCD14 antisense, is not accompanied by increased IFN-β (Fig. 7H and 7J) and in fact is accompanied by a marked suppression of IFN-β expression and secretion.

Assays of the soluble form of CD14 (sCD14) protein in CD14-antisense treated THP-1 cells after incubation with HCMV revealed depleted levels (Fig. 7K). A Western-blot for CD14 in THP-1 cells incubated with HCMV in the presence of CD14 antisense or missense confirmed a sequence-specific effect of the antisense (Fig. 7L). Together, these findings show that HCMV-induced signaling via CD14 and TLR4 is necessary for TIRAP-mediated induction of IL-8 and TRAF6-mediated activation of IFN-β.

Assays of the soluble form of CD14 (sCD14) protein in CD14-antisense treated THP-1 cells after incubation with HCMV revealed depleted levels (Fig. 7K). A Western-blot for CD14 in THP-1 cells incubated with HCMV in the presence of CD14 antisense or missense confirmed a sequence-specific effect of the antisense (Fig. 7L). Together, these findings show that HCMV-induced signaling via CD14 and TLR4 is necessary for TIRAP-mediated induction of IL-8 and TRAF6-mediated activation of IFN-β.

### Interleukin 6-mediated Induction of CD14 Leads to IL-8 and IFN-b Expression by Monocytes Incubated with HCMV

In liver cells and in the U937 and Mono-Mac-6 human myelomonocytic cell lines that are incubated with gram-negative bacteria or LPS, IL-6 is induced, and functions in an autocrine manner to induce CD14 expression [10], [21]. Therefore, we tested the possibility that IL-6 mediated similar effects after induction of CD14 gene expression in THP-1 cells incubated with HCMV. A well-characterized IL-6 neutralizing antibody down-regulated the expression of CD14 (Fig. 8B) at 6 hours of incubation with HCMV. In parallel, TIRAP (Fig. 8C), TRAF6 (Fig. 8E), IL-8 (Fig. 8F) and IFN-β (data not shown) expression levels were also strongly decreased by IL-6 antibody. Down-regulation of IL-8 (Fig. 8G), IFN-β (data not shown) and membrane-resident surface CD14 (Fig. 8H) were also detected by ELISA at 6 hours of incubation HCMV in the presence of IL-6 antibody. None of the other factors that we evaluated, i.e. TLR4 (Fig. 8A) and MD2 (data not shown) or the adaptor TRAM (Fig. 8D), decreased with IL-6 antibody treatment. Thus, we provide evidence that induction of CD14 mRNA by HCMV in THP-1 cells is mediated in part by IL-6.

### Exogenous Recombinant CD14 Rescues HCMV-induced IL-8 and IFN-b Production in IL-6 Antisense-treated THP-1 Cells

Our data indicate that HCMV-induced signaling via TLR4/MD2 appears to be important for TIRAP-mediated induction of IL-6 (Fig. 4) which in turn is required for the activation of CD14 (Fig. 8). Increased CD14 production in concert with TLR4/MD2 could promote TIRAP-mediated IL-8 expression as well as TRAF6-mediated IFN-β, possibly via TLR9 (Fig. 7). Because CD14 was reduced in the presence of IL-6 antibody (Fig. 8) and this led to a reduction of IL-8 and IFN-β (Fig. 7), a rescue-type experiment was performed with addition of exogenous recombinant CD14 after IL-6 antisense treatment to determine the potential for either a rescue effect or a supraphysiologic effect.

A concentration of 20 µg/ml of recombinant CD14 resulted in complete rescue of IL-8 (Fig. 9D and 9G) and IFN-β (Fig. 9F and 9H), suggesting that exogenous recombinant CD14 can be a key modulator in the transcription of TLR4-mediated cytokines IL-8 and IFN-β. Likewise, 20 µg/ml of recombinant CD14 rescues TIRAP (Fig. 9A) and TRAF6 (Fig. 9C) in IL-6 antisense-treated THP-1 cells Incubated with HCMV. Since TRIF has no effect (Fig. 9E), we postulate that recombinant CD14 can potentiate selected HCMV-induced innate responses in IL-6 antisense-treated THP-1 cells, through TLR4-, TLR2- and we hypothesize possibly TLR9-dependent pathways [16], [18], thereby promoting IL-8 and IFN-β activation, respectively.

## Discussion

The emerging theme from this study is the further elucidation of the relationship between HCMV and host toll-like receptor (TLR)-dependent innate immune responses. Understanding the contribution of toll like receptors and accessory proteins involved in the innate recognition of HCMV is of great interest because of the potential to identify novel intervention points for treatment strategies for cytomegalovirus infection. We previously reported that TLRs-2/-3/-9 are important cellular receptors that mediate innate immune responses and cytokine production in human monocytes incubated with Toledo strain HCMV [18]. Others had also revealed the critical role of TLR2 together with CD14 in HCMV attachment and subsequent activation of NFkB in TLR2 and CD14 over-expressing human embryonic kidney cells [16], [17]. However, questions remained with respect to other potential TLRs activated by co-incubation of innate immune cells, e.g. monocytes with HCMV. Classically, the TLR4/MD2/CD14 complex was studied in the context of LPS and viewed as a key ligand for bacterial glycolipids [2]–[4]. However, a series of experiments, including a knock-out mouse model, showed that the TLR4/MD2/CD14 complex can also be involved in the recognition and subsequent innate immune response to several viral proteins, such as vesicular stomatitis virus, mouse mammary tumor virus and respiratory syncytial virus [13]–[15].

Here, we present evidence of a previously undescribed relationship between components of the TLR4 receptor complex and HCMV in monocytes of human origin. In this study, we demonstrated that THP-1 monocytic cells strongly expressed TLR4, MD2, and CD14 after incubation with Toledo strain HCMV. We also show which pathways are involved in subsequent regulating of cytokine expression, including IL-6, IL-8 and IFN-β. In THP-1 cells incubated with HCMV, levels of TLR4 mRNA peaked early at 15 min and remained elevated throughout the 6 hours of incubation. In contrast, MD2 and CD14 responses peaked somewhat later at 1 hour and 6 hours, respectively. Our data also suggest that MD2 serves an important component in modulating IL-6 expression whereas IL-8 induction depends more on CD14. The results of this investigation suggest that HCMV was able to activate TLR4 signaling and mediate cytokine induction in human monocytic cells in a time-dependent manner and that this effect was blocked by TLR4-neutralizing antibodies. Moreover, in the presence of soluble TLR4/MD-2 receptors, there was suppression of CD14, particularly at 6 hours. We postulate that competitive interaction of HCMV with soluble TLR4/MD2 receptors prevented the process of late upregulation of CD14, implying that there was less HCMV available for interacting with cell-bound TLR4/MD2.

These findings appear in conflict with the LPS-induced TLR4/MD2 cellular activation model wherein LPS is first bound to CD14 and further interacts with the TLR4*/*MD2 complex [4], [9]. It has been suggested that CD14 is required for LPS-LBP (LPS-binding protein) binding to the TLR4/MD2 complex. So, TLR4/MD2 is thought to work downstream of this initial binding in LPS induced TLR4 signaling [4], [9]. It is possible that the CD14 membrane domain in THP-1 cells does not directly bind to an HCMV component, but that it may secondarily increase the binding to HCMV after an initial TLR4/MD2 binding takes place. Involvement of all three components (TLR4/MD2 and CD14) appears necessary as part of the HCMV-related activation which modulates IL-8 expression. These findings also revealed that the same three components appear required for initial IL-6 expression and later IL-8 expression, presumably via the TIRAP-TRAF6 pathway in THP-1 cells. In contrast, no changes in the levels of IL-6 were observed in CD14 antisense treated THP-1 cells after incubation with HCMV, suggesting that CD14 contributes minimally to IL-6 expression and release.

Another noteworthy finding was that TIRAP played a regulatory role in the HCMV-induced inflammatory cytokine response in THP-1 monocytic cells. HCMV-induced recruitment of TIRAP to TLR4 is required for induction of TRAF6 as well as expression and production of IL-6 and IL-8. However, when TIRAP was depleted by antisense, there was a large rise in TRAM expression at 6 hours. Of interest there was also a parallel increase in both TRIF and IFN-β. The high level of TRAM at 6 hours is surprisingly not accompanied by an elevation in TRAF6 expression, but rather a marked suppression of TRAF6. This phenomenon accompanying TIRAP inhibition appears to be a shift from the initial canonical MyD88-dependent pathway by which TIRAP is activated in response to HCMV. The upregulation of TRAM may be compensatory, acting as an alternative signal away from the “default” MyD88-dependent pathway when there is a deficiency of TIRAP activity. This “alternative” route appears to activate IFN-inducible genes via the TRAM-TRIF pathway. There is some support for such a concept given previously reported crosstalk between the TRAM-TRIF and TIRAP-MyD88 pathways [22].

The role of TRAM in LPS signaling has been thought to be as a bridging adapter between TLR4 and TRIF within the MyD88 independent pathway. TRAM activity has not been considered crucial for other TLR signaling. TRAM deficient mice have reportedly normal inflammatory cytokine production after ligand engagement of TLR2, TLR7 and TLR9 [2] and normal TLR4 mediated phosphorylation of IRAK, indicating that a signaling factor of the MyD88-dependent pathway that uses TRAF was also unaffected. Interestingly, our TRAM antisense results also had no effect on cytokine expression and secretion, strongly suggesting that TRAM is not be an important adaptor in the HCMV-induced canonical TLR4-mediated pathway.

CD14 expression/production has been shown by others to increase and correlate with prior IL-6 production in different inflammatory conditions of liver cells [10], and human myelomonocytic cells, such as U937 and Mono-Mac-6 cells [21]. IL-6 has been shown to regulate (increase) CD14 expression and synthesis in both HepG2 hepatocellular carcinoma cells and in human primary hepatocytes [10]. Here, we show that IL-6 is important for downstream CD14 expression and cytokine production in THP-1 monocytic cells incubated with HCMV, i.e. downregulation of CD14, IFN-b and IL-8 expression and secretion occurred after neutralizing or knocking down IL-6. These data provide the first information about the role of IL-6 in regulating HCMV-induced CD14 expression in THP-1 monocytic cells, and again show that CD14 is important for IL-8 and IFN-β induction.

Multiple lines of biochemical and genetic evidence support the contention that both soluble CD14 and GPI-anchored membrane CD14 are accessory molecules that function to enhance cellular responses to LPS [9]–[11]. No data were available to show whether sCD14 could substitute for mCD14 function in facilitating HCMV’s effects on TLR4 and MD2 in monocytes. To address this issue, we added exogenous recombinant CD14 after IL-6 antisense treatment of THP-1 cells incubated with HCMV, in order to determine the potential for rescuing IL-8 and IFN-β expression and secretion. We showed a complete rescue of TIRAP-mediated IL-8 and TRAF6-mediated IFN-β at 20 µg/ml of sCD14, indicating that CD14 is a key modulator in the transcription of TLR4/MD2-mediated cytokines IL-8 and IFN-β if IL-6 is knocked down in THP-1 cells incubated with HCMV. Since we saw that sCD14 has no effect of TRIF, we postulate that it may strongly potentiate innate response in IL-6 antisense-treated THP-1 cells, through TLR4- (current study) and/or TLR2 [16], [18] thereby promoting IL-8 and IFN-β activation, respectively.

There is a possibility that TLR9 could be involved in this CD14 related IL-8 and IFN-β production. CD14 was recently shown not only to be associated with TLR9 signaling but also engaged in the induction of type I IFN in peritoneal macrophages via the MyD88-dependent pathway [23]. Further testing in the THP-1 model would be needed to evaluate CD14 as a possible factor in the TLR9-dependent signaling pathway which is another mechanism in monocytes for the activation of TRAF6 leading to the induction of IFN-β, instead of TRIF-induced IFN-β activation. This observation adds evidence for a potential dual role of CD14 molecule in TLR2- or TLR4-induced TIRAP-mediated activation of IL-8, possibly even TLR9-induced TRAF6-mediated IFN-β activation.

There are limitations to our study. We used a single monocytic cell line so that these same responses may not be generalizable to other human monocyte populations. Future studies will be needed to explore this. We also used a single laboratory strain of HCMV. We chose Toledo strain because it retains a number of genes seen in wild type HCMV that have been lost from other laboratory strains such as AD169. Use of wild type clinical strains of HCMV also will be needed to confirm that disease producing HCMV strains induce similar responses. We use relatively short periods of incubation of THP-1 cells with Toledo strain HCMV. Thus, we are not able to differentiate the proportion of THP-1 cells that might eventually become productively infected with longer incubations. Further, we did not specifically determine the proportion of targeted THP-1 cells in which entry of HCMV or a viral component took place but did not progress, or those for which a virion or some HCMV component merely engaged the surface of the cells. The 10∶1 MOI however should allow some contact of each target THP-1 cell with HCMV. We plan studies using either glycoprotein B or UV inactivated HCMV to help understand whether non-infectious virions or even single components produce similar effects. We also did not knock down TLR2 which we and others have shown can activate in the presence of HCMV. Therefore, some of the reported effects may be due to crosstalk between TLR2 and TLR4 activation. Further double knock-down studies are thus also warranted.

Nevertheless we feel that our data add new information and raise additional questions about the innate response to HCMV and provide more understanding of importance of TLR4 and its accessory/adaptor molecules in HCMV induced monocyte responses.

## Materials and Methods

### Reagents and Kits

Dulbecco’s Modified Eagle’s Medium (DMEM), Fetal Bovine Serum, SYBR Green, Reference Dye for Quantitative PCR, and Protease Inhibitor Cocktail were obtained from Sigma-Aldrich (St. Louis, MO). RPMI Medium 1640 was obtained from Gibco/Invitrogen. RNeasy Mini Kit, Sensiscript Reverse Transcriptase Kit, QIAamp DNA Micro Kit, and QIAquick Gel Extraction Kit were all purchased from Qiagen (Valencia, CA). AmpliTaq Gold with GeneAmp 10X PCR Buffer and MgCl2 solution were from Applied Biosystems (Foster City, CA).

### Cell Culture and Treatment

Human monocytoid THP-1 cells and human foreskin fibroblasts at passage 6 to 8, purchased from ATCC (Manassas, VA), were grown in RPMI and DMEM, respectively, with 2 mM L-glutamine, 250 µg/ml amphotericin, 100 U/ml penicillin, 100 µg/ml streptomycin and 10% fetal bovine serum at 37°C under a humidified condition of 95% air and 5% CO2. Upon 90% confluence, THP-1 cells were plated at a density of ∼10^5^ cells/well in 12-well plates with serum-free RPMI overnight. On the following day, cells were either treated with serum-free medium alone or serum-free medium plus HCMV-Toledo at a multiplicity of infections (MOI) of 10 throughout the culture period. Morpholino antisense or missense control was added separately to culture media at 5 µmol/l. For inhibitory studies, soluble recombinant human TLR4/MD2 complex was added at a final concentration of 20 µg/ml with added media alone as a control. For experiments where antibody neutralized TLR4 or IL-6, 7.5 µg/ml human TLR4 neutralizing antibody or 2 µg/ml human IL-6 neutralizing antibody were use based on the manufacturer's data (R&D Systems, Minneapolis, MN) as those required to achieve >80% blocking in the blockade assay. Goat IgG antibody was added to the control cells in similar concentrations as control antibody. Freshly isolated THP-1 cells were pretreated with the antibodies for two hours and HCMV-Toledo was added to culture media at MOI of 10 for the entire culture time. In an additional experiment, exogenous recombinant human CD14 (R&D Systems, Minneapolis, MN) was added at a final concentration of 20 µg/ml, which has been established by others to enhance LPS-stimulated cytokine secretion by THP­1 cells [24]. In order to determine whether a brief incubation of THP-1 cells with HCMV results in actual infection, we co-incubated THP-1 cells and HCMV-Toledo for 1 and 6 hours followed by rinsing and subsequent incubation of cells for 3 days. After the 3 day incubation period, cells were rinsed once more, pelleted and cellular DNA was isolated. PCR was conducted on THP-1 cellular DNA using primers specific for a sequence in HCMV glycoprotein B (gB).

### Virus Preparations

Viral concentrations in the supernatant of human fibroblast cultures infected with HCMV-Toledo at 5–7 days postinfection were collected using the Millipore® (Billerica, MA) Steriflip® disposable vacuum filtration system with PVDF membrane to remove cellular debris produced during infection of fibroblasts and later using QIAamp DNA Micro Kit for the extraction of total viral DNA, following the manufacturer’s recommendation. HCMV-Toledo DNA copy number was then determined and detected by the same protocol used in the SYBR Green real-time quantitative PCR, except for the addition of HCMV primers and the HCMV-Toledo DNA sample. All virus stocks were aliquotted and stored at −80°C until used as viral inocula.

### Endo-Porter Delivery of Morpholino Antisense Oligonucleotides Assay

Morpholino antisense oligonucleotides complementary to TIRAP (5′- GGGTCAAAACTAGCCCCGTGAGACC-3′), TRAM. (5′-TTATTTTAGACTTCCCGATACCCAT-3′), CD14 (5′-CATGGTCGATAAGTCTTCCGAACCT-3′), IL-6 (5′- GGGAGATAGAGCTTCTCTTTCGTTC -3′) and missense control oligonucleotides. (5′-CCTCTTACCTCAgTTACAATTTATA-3′) were designed and synthesized by Gene Tools (Philomath, OR). Special delivery morpholino antisense oligonucleotides were transfected with Endo-Porter transfection method according to the manufacturer’s protocol. All morpholinos were complexed with Endo-Porter delivery reagent and confirmation of delivery was measured by the Western blot analysis. Note that all Endo-Porter transfection experiments were carried out in triplicate and repeated three times.

### Non-quantitative PCR

Total RNA was extracted from cells and treated with DNase. RNA was subjected to reverse transcription. cDNA was then amplified by PCR for 40 cycles (Table 1). All PCR products were separated by electrophoresis in 2% agarose gel. PCR cycles were as follows: initial denaturation at 95°C for 10 minutes, followed by 40 cycles of 94°C for 30 seconds, 60°C for 30 seconds, 72°C for 30 seconds and final extension at 72°C for 10 minutes.

### SYBR Green Real-Time Quantitative PCR

PCR amplifications were performed using a Bio-Rad iCycler (Hercules, CA) sequence detection system. Unless otherwise specified, each reaction mixture contained 10X Gold Buffer, 25 mm MgCl_2_, 2.5 mM dNTPs, 10X SYBR Green, AmpliTaq Gold polymerase, Reference Dye, dH_2_O, DNA template and 10 µM of each primer. Amplification was performed by initial polymerase activation for 10 minutes at 95°C, and 40 cycles of denaturation at 95°C for 15 seconds, annealing at 60°C for 20 seconds and elongation for 30 seconds at 72°C. The fluorescence threshold value was calculated using the iCycle system software. The calculation of relative change in mRNA was performed using the delta-delta method [25], with normalization for the housekeeping gene β-actin.

### Immunocytochemistry

Cells cultured on non-coated glass coverslips (Fisher Scientific, Pittsburgh, PA) at a density of ∼10^5^ cells/ml were fixed with 4% phosphate-buffered paraformaldehyde for 15 min at RT. After fixation, the cells were permeabilized with 0.2% Triton X-100 (Sigma, St. Louis, MO) for 5 min at RT, blocked with normal donkey serum for 30 min and then incubated with primary mouse monoclonal anti-human TLR4 antibody in a moist chamber for 2 hrs at RT. The cells were rinsed with PBS and incubated with secondary FITC-conjugated goat anti-mouse IgG for 2 hrs at RT in a dark cabinet. After several washes, coverslips containing cells were mounted onto slides in aqueous mounting medium with anti-fading agents (Biomeda Corp., Foster City, CA). Fluorescence digital images were captured using an Olympus BX60 (Melville, NY) microscope attached with Olympus U-PMTVC camera adaptor. Optronics DEI-750 (Goleta, CA) software was used to acquire and analyze images. Additionally, images (maximum intensity projection from z-stack images) were acquired using a Zeiss LSM-510 laser scanning confocal microscope at 63X magnification.

### SDS-PAGE and Western Blot Analysis

Proteins were separated on a 10% Tris-Hcl Ready Gel (Bio-Rad, Hercules, CA), transferred onto nitrocellulose membranes, and incubated with mouse monoclonal (ab6276) to β-actin at a dilution of 1/5000, rabbit polyclonal (ab96106) to TRAM at a dilution of 1/500, rabbit polyclonal (ab78070) to TIRAP at a concentration of 1 µg/ml or rabbit polyclonal (ab78313) to CD14 at a concentration of 2 µg/ml overnight at 4°C. All western blot antibodies were obtained from ABCAM (Cambridge, MA). After incubation, the membranes were washed 3X for 15 minutes in washing buffer (PBS-0.05% Tween20) and incubated with a secondary anti-rabbit (β-actin, TRAM, TIRAP and CD14) antibody coupled to horseradish peroxidase (Vector Labs, Burlingame, CA) for 1 hour at room temperature. Then, the membranes were washed 3X for 15 minutes in washing buffer, and immunoreactivity was normalized by chemiluminescence (Amersham, ECL+Plus Kit) according to the manufacturer’s instructions. The membranes were exposed to Kodak scientific imaging films (Rochester, NY) within 1 minute for detection.

### Measurement of Cytokines

To screen for HCMV-induced cytokine secretion, THP-1 cells (2.5×105) were incubated with media alone (negative control), 15 µg/mL of LPS from InvivoGen (positive control for IL-6 and IL-8), or a MOI of 10 of HCMV-Toledo. IL-6, IL-8, sCD14 and IFN-β secretion into the culture supernatants were measured by sandwich ELISA (R&D Systems) according to the protocol from the manufacturer. Data points are expressed as the mean optical density (OD) of duplicate assays at 450 nm.

### Statistical Analysis

Data were analyzed using the Microsoft Office Excel 2003 and expressed as means±S.D. where appropriate. Two group comparisons were evaluated using the unpaired Student’s t-test. Values of p<0.05 were considered statistically significant.

## Supporting Information

Figure S1 **Brief incubation of THP-1 cells with HCMV leads to productive infection.** Brief incubation of THP-1 cells with HCMV for 1 to 6 hours, followed by removal of virus and rinsing of cells, leads to HCMV infection that can be detected by standard PCR (A) or real-time PCR (B). Immunofluorescence analysis of HCMV exposed cells reveals positive immunoreactivity for both (C) immediate early antigens (IEA) and (D) late antigens (LA). Images in C and D were acquired at 63X magnification and all images are maximum intensity projection images from Z-stacks.
(TIF)
