# Supplementary figures and images for: Human Cytomegalovirus Induces TLR4 Signaling Components in Monocytes Altering TIRAP, TRAM and Downstream Interferon-Beta and TNF-Alpha Expression
Source: PLoS One. 2012 Sep 7;7(9):e44500. doi: 10.1371/journal.pone.0044500 (PMC3436894; doi:10.1371/journal.pone.0044500)

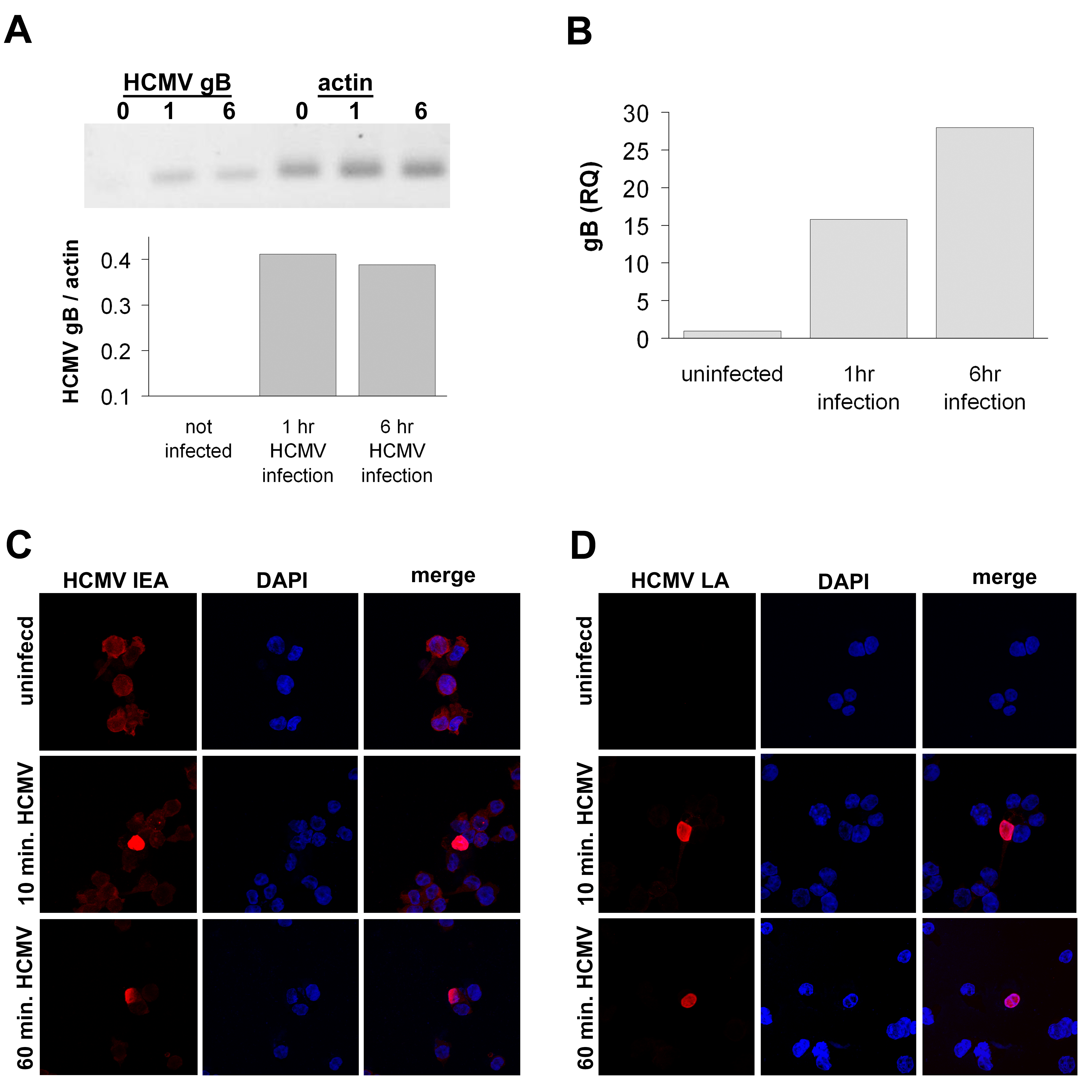

Supplement: Figure S1 — Brief incubation of THP-1 cells with HCMV leads to productive infection. Brief incubation of THP-1 cells with HCMV for 1 to 6 hours, followed by removal of virus and rinsing of cells, leads to HCMV infection that can be detected by standard PCR (A) or real-time PCR (B). Immunofluorescence analysis of HCMV exposed cells reveals positive immunoreactivity for both (C) immediate early antigens (IEA) and (D) late antigens (LA). Images in C and D were acquired at 63X magnification and all images are maximum intensity projection images from Z-stacks. (TIF) [file pone.0044500.s001.tif]
